# Aberrant Platelet Aggregation as Initial Presentation of Essential Thrombocythemia: Failure of Entero-Coated Aspirin to Reduce Platelet Hyperactivation

**DOI:** 10.3390/ijms25010176

**Published:** 2023-12-22

**Authors:** Alessandro Morotti, Cristina Barale, Michele Sornatale, Emilia Giugliano, Vittorio Emanuele Muccio, Chiara Frascaroli, Marisa Pautasso, Alessandro Fornari, Isabella Russo

**Affiliations:** 1 Department of Clinical and Biological Sciences, University of Turin, Regione Gonzole, 10, Orbassano, I-10043 Turin, Italy; alessandro.morotti@unito.it (A.M.); cristina.barale@unito.it (C.B.); michele.sornatale@edu.unito.it (M.S.); 2 Laboratory of Clinical and Microbiological Analyses, San Luigi Gonzaga Hospital, Orbassano, I-10043 Turin, Italy; e.giugliano@sanluigi.piemonte.it (E.G.); v.muccio@sanluigi.piemonte.it (V.E.M.); c.frascaroli@sanluigi.piemonte.it (C.F.); m.pautasso@sanluigi.piemonte.it (M.P.); 3 Department of Oncology, Division of Pathology, San Luigi Gonzaga Hospital, University of Turin, Orbassano, I-10043 Turin, Italy; a.fornari@sanluigi.piemonte.it

**Keywords:** essential thrombocythemia, platelet aggregation, aspirin

## Abstract

Essential thrombocythemia (ET) is a myeloproliferative neoplasm variant characterized by excessive production of platelets. Since the most common cause of mortality and morbidity in ET patients is thrombosis, the excessive production of platelets may cause thrombotic events. However, little is known about the function of platelets in ET. We report a female patient who presented as asymptomatic, without a remarkable medical history, and ET was diagnosed after an incidental finding of moderate thrombocytosis. Notably, together with thrombocytosis, an abnormal platelet phenotype was found for the presence of a massive, rapid and spontaneous formation of aggregates and platelet hypersensitivity to subthreshold concentrations of aggregating agonists. Bone marrow histopathological examination and genetic analysis with the *JAK2* (V617F) gene mutation findings confirmed the initial suspicion of ET. Although the ET patient was placed on aspirin, the persistence of the platelet hyperactivation and hyperaggregability prompted a switch in antiplatelet medication from entero-coated (EC) to plain aspirin. As result, platelet hypersensitivity to agonists and spontaneous aggregation were no longer found. Collectively, our study demonstrates that platelet function analysis could be a reliable predictor of ET and that plain aspirin should be preferred over EC aspirin to attenuate platelet hyperreactivity.

## 1. Introduction

Essential thrombocythemia (ET) is the most common type of myeloproliferative neoplasm (MPN) characterized by increased platelet counts due to abnormal thrombopoiesis in the bone marrow, which leads to the formation of platelets from the precursor cells megakaryocytes, then released into the bloodstream after detaching from the precursor cells [1]. The diagnosis of ET is based on clinical, molecular, and histopathological evaluations, with histopathology playing a major role, supported by molecular genetics. Mutated specific functions of the driver genes Janus kinase 2 (*JAK2*), calreticulin (*CALR*), and myeloproliferative leukemia (*MPL*) have myeloproliferative effects. A point mutation causing a change in the amino acid from valine to phenylalanine at codon 617 of the *JAK2* (*JAK2* V617F) gene determines a gain of function in the activity of JAK2 and the *JAK2* V617F mutation is the most frequent cause of ET, occurring in more than 50% of ET patients. With regard to platelets, it is important to remember that the number, but not the percentage, of reticulated platelets increases in ET patients without vascular disorders, whereas an increased percentage of reticulated platelets correlates with thrombotic events in ET patients with vascular disorders, thus suggesting in these subjects decreased platelet survival and increased platelet turnover [2]. However, the role of platelet function has not been well clarified, although abnormal platelet characteristics may potentially be useful in the identification and treatment of patients at high risk of thrombosis. In the latest revised International Prognostic Score for ET (IPSET)–thrombosis model, ET patients are stratified into four categories of thrombosis risk [3], and for a low-risk patients, the treatment with aspirin (acetylsalicylic acid, ASA) is recommended. However, aspirin’s effects in ET are still a matter of debate [4,5] due to the lack of a persistent platelet inhibition, which is necessary for the prevention of thrombosis.

## 2. Detailed Case Description

**Figure 1:**
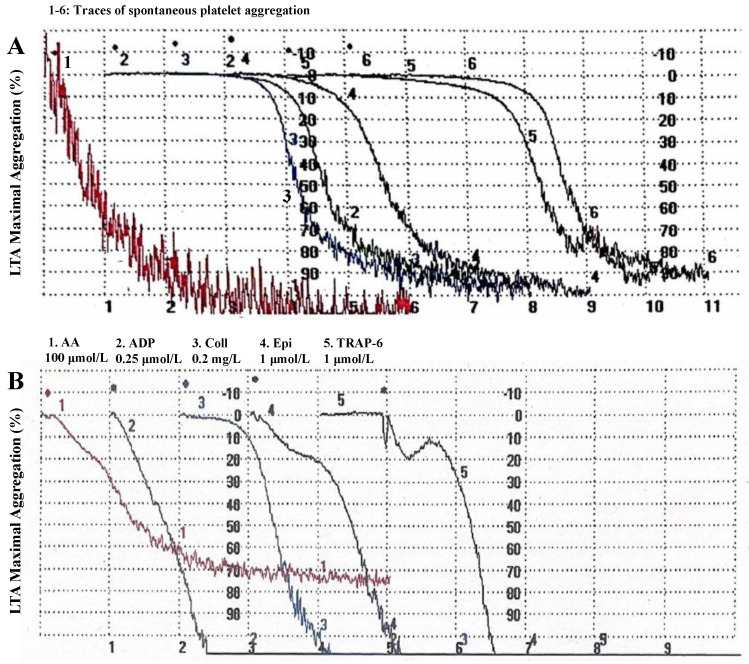
Representative light transmission aggregometry (LTA) trace analysis in platelet-rich plasma samples in the absence (spontaneous aggregation) (**A**) and in the presence (**B**) of agonists. Percent aggregation is given on the *y*-axis and time (minutes) on the *x*-axis. Abbreviations: AA, arachidonic acid; ADP, adenosine diphosphate; Coll, collagen; Epi, epinephrine; TRAP, protease-activated receptor-1 (PAR-1)–thrombin receptor activating peptide.

An asymptomatic normal weight 52-year-old woman without cardiovascular risk factors, such as smoking, diabetes, dyslipidemia, and hypertension, presented to our laboratory with mild thrombocytosis (around 500,000/µL.) Surprisingly, a massive and spontaneous, not induced, formation of platelet aggregates was observed during the preparation of platelet samples, revealing the phenomenon of spontaneous aggregation in 70% of platelet-rich plasma (PRP) samples. These spontaneous aggregations led to 100% of the maximal aggregation (MA) (Figure 1A). In the PRP samples where spontaneous aggregation was not observed, light transmission aggregometry (LTA) analysis in response to subthreshold concentrations of arachidonic acid (AA) (100 μmol/L), adenosine diphosphate (ADP) (0.25 μmol/L), collagen (0.2 mg/L), epinephrine (1 μmol/L) and protease-activated receptor-1 (PAR-1)–thrombin receptor activating peptide 6 (TRAP-6) (1 μmol/L) revealed an aggregating response ranging from 70% MA (in response to AA) to 100 % MA (to the others) (Figure 1B).

From then, she was admitted to the Hematology Unit of San Luigi Gonzaga Hospital and subjected to integrated and multidisciplinary approaches, including biochemical, hematological, immunological, and molecular analyses, to shed light on the molecular mechanisms related to the observed rare and aberrant phenotype of platelet aggregability.

### 2.1. Clinical Laboratory Determinations

To evaluate her general health status, biochemical and hematological and coagulation check-ups were performed. Complete blood count analysis revealed a normal leukocyte and erythrocyte count, normal levels of hemoglobin and hematocrit as well as a normal range of values for the first-level hemostasis analysis, including fibrinogen, D-dimer and prothrombin time (PT) and activated partial thromboplastin time (aPTT). Immunochemistry analysis did not reveal antiphospholipid antibodies, thus ruling out antiphospholipid syndrome (APS), which is one of the more common acquired causes of hypercoagulability. However, a mild increase in the platelet count (514 × 10^9^/L)) and increased values of the mean platelet volume (MPV) and platelet crit (PCT) were found (Supplementary Table S1). 

### 2.2. Genetic Tests

Based on the results of the biochemical, hematological and coagulation parameters, a diagnosis of ET was hypothesized. To confirm this hypothesis and unveil the mechanisms underlying the observed platelet abnormalities, specific genetic molecular tests were performed on venous blood. The presence of *JAK2* V617F, *JAK2* exon 12, *MPL*, and *CALR* mutations was included in the molecular diagnostic and prognostic algorithms for MPNs. The patient was negative for the B cell receptor (BCR)-Abelson murine leukemia 1 (*ABL1*) translocation, referred to as the Philadelphia chromosome. No variants were found in *MPL*, in *CALR*, nor *JAK2* exon 12, while the *JAK2* V617F variant was identified (Supplementary Table S1).

### 2.3. Differential Diagnosis

Since the *JAK2* V617F mutation was identified during the blood tests, leading to a suspicion of MPN, a bone marrow biopsy was performed. Genetic analysis carried out on bone marrow aspiration confirmed that the patient had the *JAK2* V617F mutation (8.5% allele burden). Flow cytometric analysis of the bone marrow aspiration at admission aimed at verifying the maturation and number of common myeloid progenitors originating from hematopoietic stem cell progenitors revealed no particular alterations in terms of the blasts’ percentage and maturation of granulocytes, monocytes and B lymphocytes (Supplementary Figure S1). The histopathological analysis revealed normocellular bone marrow with only mild megakaryocytic hyperplasia; some enlarged megakaryocytes containing multilobed nuclei with a focal tendency to form loose clusters were identified (Supplementary Figure S2). The positivity for the *JAK2* V617F mutation in the marrow blood genetic analysis also contributed to the diagnosis of ET. According with the risk stratification for thrombosis, the patient was prescribed low-dose entero-coated aspirin (EC-ASA) (100 mg/day) as a prophylactic anti-thrombotic therapy.

### 2.4. Aspirin’s Effects on Platelet Aggregation

#### 2.4.1. Platelet Response to In Vivo Treatment with EC-ASA

**Figure 2:**
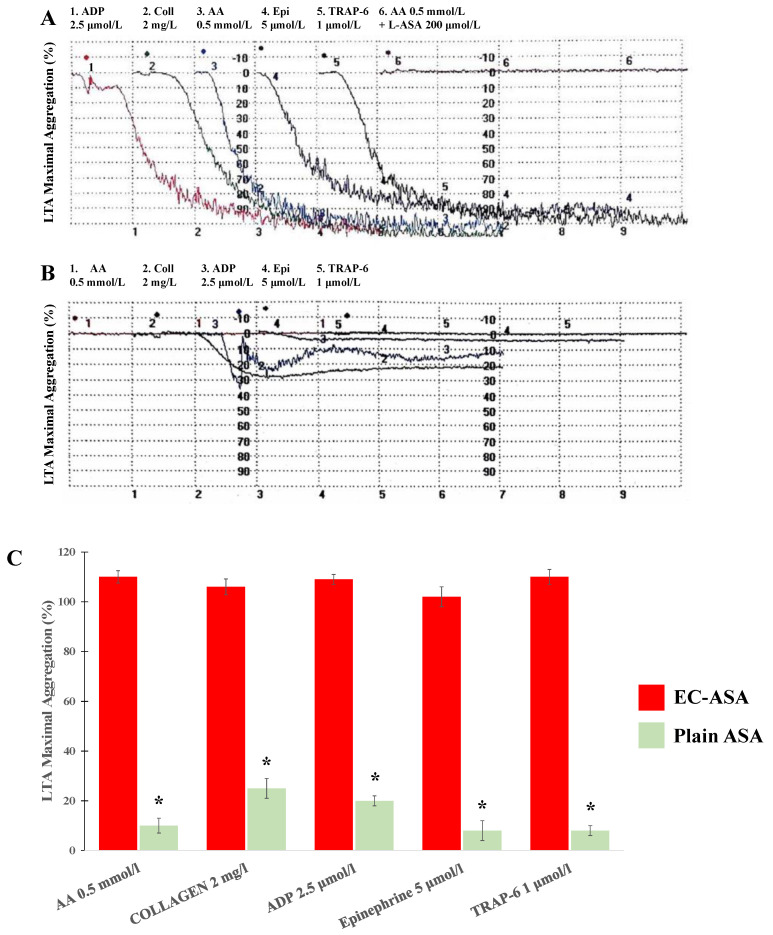
Representative light transmission aggregometry (LTA) trace analysis in platelet-rich plasma samples stimulated by agonists after in vivo treatment with entero-coated aspirin (EC-ASA) (**A**) or plain aspirin (plain ASA) (**B**). Percent aggregation is given on the *y*-axis and time (minutes) on the *x*-axis. Histogram showing the LTA maximal aggregation percent values (mean ± SEM) after either EC-ASA or plain ASA treatment (**C**). Plain ASA vs EC-ASA: * *p* < 0.0001. Abbreviations: AA, arachidonic acid; ADP, adenosine diphosphate; TRAP, protease-activated receptor-1 (PAR-1)–thrombin receptor activating peptide.

At follow-up 5 days, 3 weeks and 2 months later, platelet aggregation analyses in the ET patient were monitored. Despite a regular EC-ASA (100 mg/day) intake, spontaneous aggregations with 100% MA were surprisingly noticed in 13–50–100% of the PRP samples, respectively. In the samples without spontaneous aggregations, LTA analysis revealed a complete absence of the in vivo inhibitory action of EC-ASA on cyclooxygenase 1 (COX-1) activity, as demonstrated by hyperaggregability in response to 0.5 mmol/L AA (Figure 2A). However, a 10 min preincubation in vitro with lysine acetylsalicylate (L-ASA) (200 μmol/L) completely blunted the AA effects on platelet aggregation (Figure 2A). Thus, on the one hand, these data suggest an absent platelet response to inhibitory effects of EC-ASA in vivo and, on the other hand, a normal platelet sensitivity to the pharmacological action of ASA in vitro. LTA induced by the other agonists still revealed a pronounced sensitivity to aggregability, given that we found 100% MA to collagen (2 mg/L), ADP (2.5 μmol/L), epinephrine (5 μmol/L), and TRAP-6 (1 μmol/L) (Figure 2B).

#### 2.4.2. Platelet Response to In Vivo Treatment with Plain ASA

After a 2-month treatment with EC-ASA, the patient was switched to plain, uncoated-ASA. After a 3-day treatment with plain ASA (100 mg/day), spontaneous aggregation was also no longer present and platelet hypersensitivity to subthreshold doses of agonists was no longer detected. Platelet aggregation was inhibited in response to 0.5 mmol/L AA (MA < 4%), 2 mg/L collagen (MA < 30%), 2.5 μmol/L ADP (MA < 20%), 5 μmol/L epinephrine (MA < 5%), and 1 μmol/L TRAP-6 (MA < 50%) (Figure 2B). Unequivocally, the comparison between the effects of EC-ASA and plain ASA on agonist-induced platelet aggregation clearly show the failure of EC-ASA to reduce platelet reactivity to agonists and the expected favorable inhibition of the platelet response exerted by plain ASA (Figure 2C). 

## 3. Discussion

In this study, an aberrant platelet tendency to aggregate in ET at diagnosis and a favorable anti-aggregating profile of plain aspirin instead of the enteric-coated aspirin were observed.

The main causes of morbidity and mortality in patients affected by ET consist of an increased risk of arterial and, more rarely, venous thromboembolic events, thrombosis and bleeding manifestations [6]. In fact, thrombotic complications in patients with MPN may involve the arterial and/or the venous system. Most arterial thrombotic events generally involve the cerebrovascular, peripheral vascular, coronary arterial or the microvascular systems. Venous thrombosis occurs in 25–40% of patients and may involve large vessels such as the portal vein and inferior vena cava [6]. Since ET is characterized by persistently elevated platelet counts and an increased risk of thromboembolic events, abnormal platelet function may potentially be useful in the identification and treatment of patients at high risk of thrombosis. Spontaneous aggregation in ET patients has been described by very few studies [7,8], and no study so far has reported this phenomenon as massive as that observed in our case. 

The agonist-induced platelet aggregation in ET patients was evaluated in studies [9] that used different methods and different agonists. These investigations reported controversial results; most of them showed a reduced platelet response to agonists, while just in one study higher aggregation values were found in ET patients in comparison with healthy controls. Others reported the abnormal aggregation without stating whether it was increased or decreased, maybe because these studies are quite old. *JAK2* V617F seems to not affect the in vitro aggregation response of ET platelets to agonists [10]. Collectively, there is agreement on increased platelet activation, especially among ET patients with a history of thrombosis, but the results regarding platelet aggregation are still inconclusive. Since platelet activation is, at least partly, dependent on the disease mutational background, it remains to be established whether platelet activation represents an independent thrombosis risk factor. However, this may be contrasted with evidence of reduced platelet aggregation in some patients, sometimes as result of acquired von Willebrand disease.

Here, we describe the case of a 52-year-old woman who presented as asymptomatic, without cardiovascular risk factors and autoimmune/inflammatory diseases, in whom ET was diagnosed as an incidental finding related to ex vivo abnormal platelet reactivity. In particular, a massive, rapid and spontaneous formation of aggregates with LTA MA values also higher than 100% occurred in PRP samples obtained from an incidental venous blood withdraw. Furthermore, the increased spontaneous aggregation observed also in the whole blood and platelet hypersensitivity to subthreshold concentrations of different types of agonists in the PRP and whole blood samples, where spontaneous aggregation did not occur, confirmed a platelet phenotype of high hyperreactivity. Genetic analyses of the whole blood and bone marrow aspiration revealed the presence of the *JAK2* V617F gene mutation and, meanwhile, bone marrow biopsy demonstrated some mature enlarged megakaryocytes with multilobed nuclei, focally organized in loose clusters. Thus, ET was diagnosed. The *JAK2* V617F mutation is considered a crucial factor in the clinical course of ET. The risk of thrombosis almost doubles with the presence of this mutation. *JAK2* mutations are also considered to be an independent risk factor for splanchnic vein thrombosis from MPN [11]. Notwithstanding, the role of platelets in arterial/venous thromboembolism associated with the *JAK2* mutation in ET remains to be elucidated. In particular, 20 out of 21 studies reported that, although in ET platelets are activated, their aggregation was reduced [9]. Intriguingly, in our patient, a dramatic hyperaggregability, spontaneous or induced, was evident at her first presentation, also confirmed at subsequent blood sample collections up to the intake of plain aspirin. In light of this, an important question relates to the mechanisms causing the hyperaggregability of platelets in our patient. One hypothesis is that the abnormal megakaryocyte clone with more sequence variants or mutations might produce platelets unusually hypersusceptible to activation and aggregation but sensitive to inhibitors. 

In our case, the abnormal platelet aggregability was the primum movens leading to the subsequent diagnosis of ET. Once diagnosed, a very important step is to risk stratify patients. The patient in this study was categorized as low risk, guiding healthcare professionals to prescribe EC aspirin, the most widely used aspirin formulation. 

### ET and Aspirin

Aspirin is the recommended treatment for the prevention of vascular events in all the ET risk categories. However, its effect in ET is still a matter of debate [4,5]. In many cases, ET patients treated with a once-daily aspirin regimen show a lack of inhibition of platelet thromboxane A2 (TXA2) synthesis persisting for 24 h, which is necessary for the prevention of thrombosis. A possible explanation for this incomplete inhibition of COX-1 activity is that the daily platelet production is increased, leading to the circulation of a high number of immature (non-acetylated COX-1) platelets. Bearing in mind that the recovery of the ability to synthesize ex novo TXA2 after aspirin ingestion in ET is a function of the platelet count [12], it is not surprising that this problem was overcome by twice daily administration of aspirin [13]. Of course, when the number of platelets is normalized, the regimen twice a day, which would expose patients to a potential and unnecessary increase in the risk of gastrointestinal side effects, would no longer be necessary. The poor responsiveness (“resistance”) to aspirin mainly referred to the administration of the entero-coated formulation of aspirin remains an open question concerning its effectiveness in platelet inhibition and clinical protection from thrombosis. 

Up to the 2-month follow-up with EC aspirin (100 mg/day), no resolution of the platelet abnormalities was observed in our case; spontaneous aggregation continued to be seen, and the MA values of the platelet aggregation curves both in the absence and in the presence of agonists were higher than 100% in response to each agonist.

This poor responsiveness (“resistance”) to EC aspirin in our case of ET could initially be explained by the increased platelet number and turnover in ET patients, which cause the release of newly formed immature platelets unexposed to aspirin and thus manifesting as high on-treatment platelet reactivity [14,15], even though she had a mild thrombocythemia.

Indeed, a systematic review of 24 observational studies on ET showed a modest reduction in thrombotic events from aspirin therapy [16] and the incomplete ability of EC aspirin to inhibit TXA2 biosynthesis was a possible explanation leading to insufficient protection from thrombosis [17]. It is important to remember that EC aspirin, absorbed in the small intestine, was developed to reduce the risk of gastrointestinal discomfort, mucosal ulcerations and bleeding exerted by plain aspirin [18], which is absorbed in the stomach. Indeed, compared with plain aspirin, the pharmacokinetics of EC aspirin was demonstrated to be less effective on platelets due to the lower ability to inhibit platelet TXA2 biosynthesis [19]. Furthermore, EC aspirin has been demonstrated to not be safer than plain aspirin in preventing gastrointestinal bleeding and ulcers, which are likely caused by the systemic effects of the drug, thus disappointing the expectations [2].

The persistence of the platelet hyperactivation and hyperaggregability prompted a switch in the antiplatelet medication to plain ASA. Just a follow-up after 3 days with plain aspirin led to platelet inhibition. Specifically, platelet hypersensitivity to subthreshold doses of agonists was no longer detected, and spontaneous aggregation was also no longer seen. As we expected for this drug, a greatly decreased platelet aggregation response was evident. Therefore, plain aspirin had a more favorable pharmacological profile than EC aspirin and the current platelet aggregation profile of our patient suggests that the thrombotic risk could be now significantly reduced.

## 4. Methods

### 4.1. Clinical Laboratory Determinations

Blood samples were taken in the morning (8:00 a.m.). Standard biochemical and immunological analyses were performed using a standard serum and plasma analyzer via automated chemistry in the central laboratory of our hospital. All the procedures took place according to the manufacturer’s recommendation.

#### 4.1.1. Molecular Analyses

The real-time quantitative polymerase chain reaction (qPCR) technique was performed to detect and quantify the mutation *JAK2* V617F (Mutaquant CE-IVD Ipsogen kit, Taqman system, Qiagen, Hilden, Germany) using the Rotorn Gene Q (Qiagen, Hilden, Germany); the t(9;22)(q34;q11) *BCR-ABL* rearrangement (*BCR-ABL* fusion kit, Diatech Pharmacogenetics, Jesi (AN) Italy) using the EasyPGX system (Diatech Pharmacogenetics, Jesi (AN) Italy); and the W515L/K *MPL* gene mutation (MutaScreen Ipsogen kit, Qiagen, Hilden, Germany) using the Rotor Gene Q (Qiagen, Hilden, Germany). Capillary electrophoresis was carried out for the qualitative analysis of *JAK2* exon-12 (Invitrogen *JAK2* exon-12 F&R primer, Thermo Fisher Scientific, Waltham, MA, USA) and *CLR* exon-9 (Invitrogen *CLR* exon-9 F&R primer, Thermo Fisher Scientific, Waltham, MA, USA) gene mutations using the AbiPrism 3500 XL DX (Thermo Fisher Scientific, Waltham, MA, USA). All the genetic molecular analyses were carried out in the central laboratory of our hospital.

#### 4.1.2. Differential Diagnosis

Since the *JAK2* V617F mutation was identified during blood tests, leading to a suspicion of MPN, a bone marrow biopsy was performed. Genetic analysis carried out on the bone marrow aspiration confirmed that the patient had the *JAK2* V617F mutation (8.5% allele burden). Flow cytometric analysis of the bone marrow aspiration at admission aimed at verifying the maturation and number of common myeloid progenitors originating from hematopoietic stem cell progenitors revealed no particular alterations in terms of the blasts’ percentage and maturation of granulocytes, monocytes and B lymphocytes (Supplementary Figure S1). The histopathological analysis revealed a mild megakaryocytic hyperplasia with a low degree of pleiomorphism and some enlarged megakaryocytes containing multilobulated nuclei with a tendency to form focal loose clustering (Supplementary Figure S2). The positivity for the *JAK2* V617F mutation in the marrow blood genetic analysis also contributed to the diagnosis of ET. According to the risk stratification for thrombosis, the patient was prescribed low-dose entero-coated aspirin (EC-ASA) (100 mg/day) as a prophylactic anti-thrombotic therapy.

The differential diagnosis included other causes of clonal neoplasms and reactive thrombocytosis. Clonal causes included different types of MPN, including PV and PMF, that can overlap considerably. Therefore, to be sure of the diagnosis of ET, it is important to rule out the other myeloproliferative disorders. If there is a suspicion of MPN, a bone marrow biopsy is strongly recommended in order to perform leucocytes immunophenotyping in marrow blood and histopathological and immunohistopathological analyses of the bone marrow.

#### 4.1.3. Leucocytes Immunophenotyping

Immunophenotyping of leucocytes was performed via multiparametric flow cytometry (FC) analysis carried out at the central laboratory of San Luigi Gonzaga Hospital. 

The bone marrow aspirate followed the lyse–wash–stain method. Briefly, 50 μL of fresh sample was dispensed in three tubes, respectively, processed via BD FACS Lyse Wash Assistant and stained with two eight-color lyotubes, designed following EUROFLOW panels, added with other antibodies as follows: CD16 FITC/CD13 PE/CD34 PerCP-cy5.5/CD117 PE-Cy7/CD11b APC/CD10 APC-H7/HLA-DR 450/CD45 V500/CD56 APC-R700/ CD64 BV605/CD33 BV711/CD64 BV786; CD36 FITC/CD105 PE/CD34 PerCP-cy5.5/CD117 PE-Cy7/CD33 APC/CD71 APC-H7/HLA-DR 450/CD45 V500/CD38 APC-R700/ CD45RO BV605/CD45RA BV711/CD15 BV711; CD20 FITC/CD10 PE/CD19 PerCP-cy5.5/CD45 V500. All the antibodies were produced by BD (Becton Dickinson, San Jose, CA, USA). Data acquisition was performed on a BD FACS Lyric (BD). For each sample, 100,000 total events were collected for analysis. Leucocytes were identified as CD45-positive cells. The data acquired were analyzed using BD FACSuite, Version 1.5 Software (BD).

#### 4.1.4. Histopathological Analyses

Bone marrow aspirate samples were obtained from the posterior iliac crest and analyzed by the Pathology Unit of San Luigi Gonzaga Hospital. Aspirated bone marrow was smeared directly onto glass for morphologic assessment after staining. An additional 0.5 to 1 mL aliquot was collected into EDTA anticoagulated vacutainers and processed within 2 h of collection. A bone marrow trephine biopsy specimen was collected, formalin-fixed and paraffin-embedded using standard methods. Then, 5 μm thick sections were cut and stained with hematoxylin and eosin; additional Giemsa staining and Gomori silver staining for reticulin fibers were performed, along with immunohistochemical reactions for MPO, CD71, CD61 and CD34. Immunohistochemistry was essential in order to correctly quantify the erythroid, myeloid and megakaryocytic elements as well as the amount of CD34-positive blasts. Histological diagnosis of ET was based on the 2016 WHO classification criteria. 

### 4.2. Platelet Studies

#### 4.2.1. Preparation of Platelet Samples

Venous blood samples were collected without stasis and with the anticoagulant 3.2% trisodium citrate (0.109 mol/L) at pH 7.4 (*v*/*v*: 1/9) for platelet aggregation studies in PRP samples. PRP for optical aggregation test was obtained using the platelet function centrifuge (BioData Corporation, Horshman, PA, USA) designed to provide rapid separation of PRP via centrifugation for 30 s. From the top, only two-thirds of the supernatant were collected to avoid contamination by other circulating cells and the remaining blood was further centrifugated for 180 sec to obtain platelet-poor plasma (PPP). 

#### 4.2.2. Platelet Aggregation Analysis

LTA is considered an ideal, relatively simple, and reliable method to specifically determine platelet activity. For these reasons, LTA is a gold standard assay for platelet function [20]. Samples of PRP were transferred into a cuvette containing a stir bar and the PRP cuvettes placed into heating slots for 2 min to warm up to 37 °C. The evaluation of platelet aggregation in the PRP was performed with the Platelet Aggregation Profiler equipped with PAP-8E channels (BioData Corporation, Horsham, PA, USA) under constant magnetic stirring at 900 rpm according to the Born method [21]. Initially, the light transmission percentage was set at 100% with a PPP sample. Each aggregation was measured as the reduction in absorbance across the sample. To define the role of various platelet membrane receptors and/or signaling pathways in platelet aggregation, a range of physiological agonists (Mascia Brunelli, Milan, Italy), including AA (TXA2R), ADP (P2Y1 and P2Y12), collagen (glycoprotein VI [GPVI] and α2β1), epinephrine (α2A), or TRAP-6 (PAR-1), were used. In the PRP samples, we also evaluated the effect of a 15 min preincubation with L-ASA (200 μmol/L) (Sanofi-Aventis, Milan, Italy), which is a soluble salt converted into ASA. We preferred L-ASA to ASA because it is soluble in water instead of in dimethyl sulfoxide (DMSO) or ethanol, which influences platelet function [22]. Platelet aggregation in response to agonists was reported as the MA. Each aggregation test was recorded for 5 min and replicated four times.

## 5. Conclusions

In conclusion, the case reported herein suggests that in ET functional studies of platelets, hemostasis analysis could be useful to stratify and explore the causes leading to thrombosis. Indeed, the peculiarity of this study was that the abnormal entity of spontaneous aggregation and hyperaggregability to subthreshold agonist concentrations in an asymptomatic subject led to diagnosis of ET. Furthermore, we were able to follow the change that occurred with respect to the differences in the platelet activation profile seen during treatment first with EC aspirin and then with plain aspirin. Since the goal of ET treatment is the prevention of thrombosis and bleeding, which are leading causes of morbidity and mortality, monitoring of the antiaggregating effects of aspirin may help to adjust anti-platelet therapy, given that it is known that the presence of circulating activated, and pro-aggregating platelets correlates with an increased thrombotic tendency. The rarity of thrombotic events in patients with normal or hypoactive platelets also supports the usefulness of platelet function analysis studies in ET. With this insight, it seems appropriate to recommend low-dose plain aspirin in patients with hyperactive platelets, even in the absence of clinically evident thrombotic events. 

## Data Availability

Data are contained within the article and Supplementary Materials.

## Supplementary Materials

The supporting information can be downloaded at https://www.mdpi.com/article/10.3390/ijms25010176/s1.
