# Supplementary material for: Aberrant Platelet Aggregation as Initial Presentation of Essential Thrombocythemia: Failure of Entero-Coated Aspirin to Reduce Platelet Hyperactivation
Source: Int J Mol Sci. 2023 Dec 22;25(1):176. doi: 10.3390/ijms25010176 (PMC10778871; doi:10.3390/ijms25010176)
Supplement: Supplementary file 1 [file ijms-25-00176-s001.zip › ijms-2775821-supplementary.pdf]

BLOOD COUNT	VALUE	U.M.	REFERENCE RANGE
**Leukocytes**	6,93	x1000/uL	[4.5-11.0]
**Erythrocytes**	4,48	x1000000/uL	[4.0-5.0]
**Hemoglobin**	13,3	g/dL	[12.0-15.5]
**Hematocrit**	41,4	%	[35.0-47.0]
**Mean Corpuscular Volume (MCV)**	92,3	fL	[80-100]
**Mean Corpuscular Hemoglobin (MCH)**	29,7	pg	[27.0-31.0]
**Mean Corpuscular Hemoglobin Concentration (MCHC)**	32,2	g/dL	[33.0-37.0]
**Red blood cells Distribution Width (RDW)**	15,1	%	[11.0-15.0]
**Platelets**	514	x1000/uL	[150-450]
**Platelet Distribution Width (PDW)**	14,2	%	[15.0-65.0]
**Plateletcrit (PCT)**	0,60	%	[0.12-0.36]
**Mean Platelet Volume (MPV)**	11,2	fL	[7.2-11.1]
**Neutrophils%**	53,9	%	[40.0-74.0]
**Lymphocytes%**	32,4	%	[19.0-48.0]
**Monocytes%**	9,0	%	[3.4-9.0]
**Eosinophils%**	3,6	%	[0.0-7.0]
**Basophils%**	1,1	%	[0.0-1.5]
**Neutrophils#**	3,74	x1000/uL	[1.90-8.00]
**Lymphocytes#**	2,24	x1000/uL	[0.90-5.20]
**Monocytes#**	0,62	x1000/uL	[0.16-1.00
**Eosinophils#**	0,24	x1000/uL	[0.00-0.80]
**Basophils#**	0,07	x1000/uL	[0.00-0.20]
**Reticulocytes**<br>**Immature Reticulocyte Fraction (IRF)**<br>**Reticulocytes%**<br>**Reticulocytes#**	 0,102<br>1,62<br>72,50	 <br>%<br>x 1000/mcL	 <br>[0.5-2.0]<br>[25-75]
**Prothrombin Time (PT)**<br>**PT (Ratio)**	1,01<br>1,01	INR	[0.80-1.20]<br>[0.80-1.20]
**Activated partial thromboplastin time (aPTT)**<br>**aPTT (Ratio)**	32,40<br>1,09	Seconds<br>Ratio	[22,00-34,00]<br>[0.80-1.25]
**Fibrinogen**	338	mg/dL	[150-400]
**Glucose**	83	mg/dL	[70-110]
**Creatinine**<br>**Glomerular Filtration Rate (GFR)**	0,79<br>76	mg/dL	[0.60-1.30]
**Aspartate Aminotransferase (AST)(GOT)**	21,0	U/L	[10.0-42.0]
**Alanin Aminotransferase (ALT)(GPT)**	20,0	U/L	[10-50]
**Bilirubine Fractionated:**<br>Total Bilirubine<br>Direct Bilirubine<br>Indirect Bilirubine	 0,80<br>0,27<br>0,52	 mg/dL<br>mg/dL<br>mg/dL	 [0.10-1.00]<br>[0.00-0.35]<br>[0.00-0.65]
**Lactate dehydrogenase (LDH)**	288	U/L	[125.00-243.00]
**Homocysteine**	8,45	mcmol/L	[<13.0]
**Ab LAC**<br>**aPTT LA screening**<br>**Dilute Russell Viper Venom Test (DRVVT)**	Negative<br>1,05<br>0,89	 Ratio<br>Ratio	 [<1.28]<br>[<1.24]
**Ab anti-phospholipid:**<br>ab anti cardiolipin IgG<br>ab anti cardiolipin IgM<br>ab anti beta2 glycoprotein IgG<br>ab anti beta2 glycoprotein IgM	Negative<br>1,70<br>2,30<br>0,70<br>2,90	 GPL/ml<br>MPL/ml<br>U/ml<br>U/ml	 [<11.0]<br>[<30.0]<br>[<9.0]<br>[<9.0]
**Molecular and cytogenetic analyses (peripheral blood):**<br>molecular analysis *BCR-ABL* rearrangement t(9;22)(q34;q11)<br>quantitative molecular analysis of *JAK2* V617F mutation<br>molecular analysis of *JAK2* (exon 12)<br>molecular analysis of *MPL* W515L/K mutation<br>molecular analysis of *CALR*	 Negative<br>6,1<br>Negative<br>Negative<br>Negative		 <br><br>[<0,1]<br><br>[<0,1]

Supplementary Table S1. Biochemical, immunological, genetic analysis on peripheral venous blood of patient.

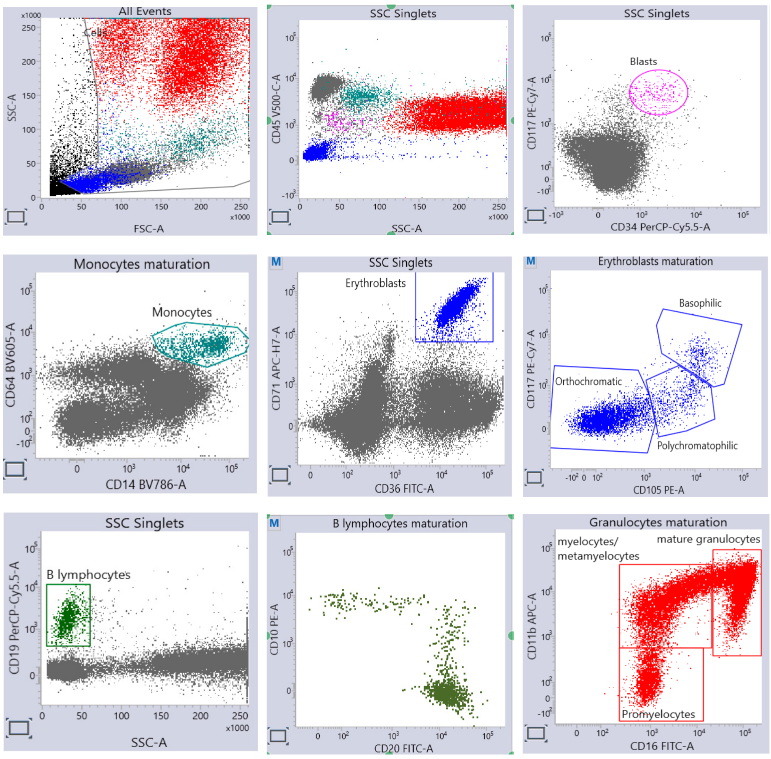

Supplementary Figure S1. Leucocytes Immunophenotyping by flow cytometry analysis carried out on bone marrow aspirate.

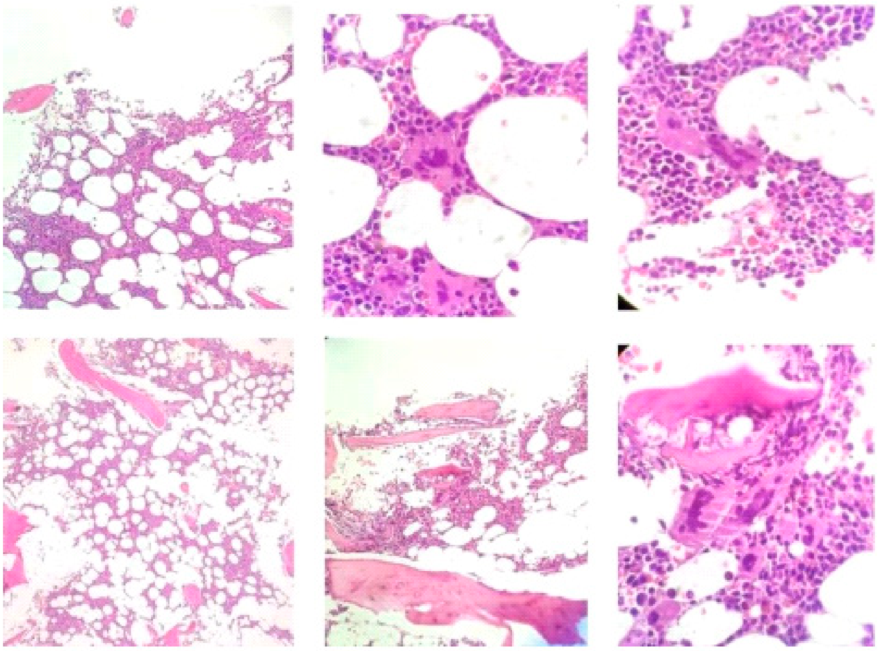

Supplementary Figure S2. Histological analysis of bone marrow. Microscope magnifications: upper left and lower left 100x, central bottom 200x, central top, upper right and lower right 400x
